# Facile Fabrication of Fluorescent Inorganic Nanoparticles with Diverse Shapes for Cell Imaging

**DOI:** 10.3390/nano9020154

**Published:** 2019-01-26

**Authors:** Guifang Wang, Jing Wang, Linlin Zhao, Qiang Zhang, Yan Lu

**Affiliations:** School of Materials Science & Engineering, Tianjin Key Laboratory for Photoelectric Materials and Devices, Key Laboratory of Display Materials & Photoelectric Devices, Ministry of Education, National Demonstration Center for Experimental Function Materials Education, Tianjin University of Technology, Tianjin 300384, China; 18202669886@163.com (G.W.); luxingzhao@hotmail.com (L.Z.); zdianq2007@126.com (Q.Z.)

**Keywords:** inorganic nanoparticles, shape, cell imaging, fluorescent probes

## Abstract

In the present work, we describe a facile and general method of fabricating fluorescent inorganic nanoparticles with diverse shapes for cell imaging application. The hematite (α-Fe_2_O_3_) nanoparticles (HNPs) with three different shapes (i.e., spindle shape, ellipsoidal shape and quasi-spherical shape) were first prepared as model systems in consideration of good biocompatibility and the controllable morphology of α-Fe_2_O_3_. Three fluorescent HNPs with different shapes were readily achieved via one-pot sol-gel reaction of AIE luminogen-functionalized siloxane (AIEgen-Si(OCH_3_)_3_) and TEOS in the presence of PVP-stabilized HNPs. Due to the fluorescence originating from the thin AIEgens-contained SiO_2_ shell around the HNPs, their photoluminescent intensities can be tuned by changing the concentrations of TEOS and AIEgen-Si(OCH_3_)_3_ in feed prior to the sol-gel reaction. When the as-prepared fluorescent products were dispersed in water, they gave intense green light emission upon excitation at 360 nm with relatively high fluorescence quantum yield. Further, fluorescent HNPs exhibited low cytotoxicity and excellent photostability and, thus, were used as optical probes to preliminarily explore the effect of nanoparticle shapes on their cellular uptake behaviors. This work should open a facile way to prepare various fluorescent inorganic nanoparticles with specific morphology for various biological applications.

## 1. Introduction

Since the clinical application of nanoparticles (NPs) for cancer drug delivery was approved in 1995, various NP-based delivery platforms were developed to break through the biological barrier to achieve drug delivery effectively. In the process, it was demonstrated that NP shape has a great influence on their interaction with biological systems, including cellular uptake, plasma circulation, and organ distribution and so on [1,2,3,4]. At present, the popular materials for fabrication of NPs for biological applications mainly include inorganic materialsre [5,6,7,8,9,10,11,12,13], polymers [14,15], viruses [16] and bacteria [17] etc. Among them, various inorganic materials, for example, Au [5,6], silica [7,8,9,10], iron oxide [11,12] and titanate [13] have received intense attention due to relative ease of synthesis, controllable morphologies and good biocompatibility. Hence, inorganic NPs should be an ideal model system to investigate the relationship between NPs’ shape and their interaction with biological systems. However, it is difficult to conduct these researches directly and visibly because most of inorganic NPs themselves are non-luminescent. Recently, fluorescence probes have shown their unique advantages including low-cost, high sensitivity, and high temporal and spatial resolution, which are especially suitable for in vivo bio-imaging [18,19,20]. So, the development of a facile and general method for preparing fluorescent inorganic NPs with different shapes is highly desired for understanding the role of their shapes in the interactions with biological species in a non-intrusive and visual ways.

Many efforts have been made to construct fluorescent inorganic NPs with controlled size and morphology, good colloidal stability, high monodispersity and high fluorescence quantum yield in a physiological environment [19]. Among these pioneering studies, various organic dyes such as rhodamines [21,22,23], fluoresceins [24,25], BODIPYs [26,27] and coumarins [28], etc. have been doped into inorganic NPs to provide desired fluorescence for cellular labeling or imaging. These hybrid NPs generally gave rather weak emission because of the significant quenching caused by the aggregation of dyes in the solid state. To address this problem, luminogens with the feature of aggregation-induced emission (AIEgens), were introduced into the fabrication of fluorescent inorganic NPs via non-covalent or covalent binding, leading to enhanced fluorescence brightness and superior photostability [29,30,31,32,33,34]. For example, Prasad et al. firstly reported AIEgens functionalized SiO_2_ NPs via a physical doping method [29]. Tang et al. prepared AIEgens-doped composite SiO_2_ nanospheres which contained aggregates of several smaller magnetite NPs via the sol-gel reaction [32]. Yu’s group synthesized (AIEgens)-functionalised mesoporous silica nanoparticles through post grafting AIEgens onto mesoporous silica nanoparticles [35,36]. They also used the AIEgens (tetraphenylethylen-containing organosilica precursor) as a silicon source to fabricate fluorescent mesoporous silica. Then, the cyclodextrin-modified CuS was assembled onto the AIEgen-containing mesoporous silica nanoparticles to act as a cell imaging agent and photothermal agent [37].

Unlike the reported work, we developed a facile and general way to prepare fluorescent inorganic nanoparticles with diverse shapes by a strategy of covalently immobilizing AIEgens within a thin SiO_2_ shell on the surface of inorganic nanoparticles with a special shape for the purpose of studying the role of nanoparticle shapes on the interaction with living cells, especially cellular uptake behaviors. In this strategy, hematite nanoparticles were chosen as model systems to provide templates for the fabrication of their corresponding fluorescent products on account of controllable morphology, easy surface modification and low-cost. AIEgen-functionalized siloxane (AIEgen-Si(OCH_3_)_3_), rather than other similar derivatives, was used based on its advantage in simple synthesis, adjustable structure, and facile sol-gel reaction with TEOS to form silica shells with good biocompatibility on the surface of nanaparticles.

Three fluorescent hematite (α-Fe_2_O_3_) nanoparticles (HNPs) with diverse shapes (i.e., spindle shape, ellipsoidal shape and quasi-spherical shape) were easily prepared by a one-pot sol-gel reaction of AIEgen-Si(OCH_3_)_3_) and TEOS on the surface of PVP-coated HNPs with the purpose of exploring the role of NP shapes on the interaction with living cells, especially cellular uptake behaviors. Three hybrid products exhibited intense fluorescence, good colloidal stability, excellent photostability, and low toxicity in vitro. Time-dependent cell imaging experiments were carried out, respectively, using three fluorescent HNPs. The results preliminarily demonstrate that NP shape has a significant influence on cellular endocytosis, and the ellipsoidal HNPs show a higher cellular uptake rate compared to those of spindle and quasi-spherical ones.

## 2. Experimental Section

### 2.1. Materials and Instrument

Ferric nitrate nonahydrate (Fe(NO_3_)_3_·9H_2_O, ≥98.5%), sodium dihydrogen phosphate (NaH_2_PO_4_, >99%) and sodium azide (NaN_3_, >99%) were purchased from local suppliers. AIEgen-Si(OCH_3_)_3_ was synthesized according to the literature [31]. Polyvinyl pyrrolidone (PVP, average *M*_w_ = 58,000), 3-chloropropyltrimethoxysilane (98%) were provided by Alfa Aesar. 1,1,2,2-tetrakis(4-ethynylphenyl)ethene (97%) was provided by Alfa Chem. Co., Ltd. (Zhengzhou, China). Tetraethyl orthosilicate (TEOS, >98%) and bromotris(triphenylphosphine)copper(Ӏ) (98%) were obtained from Aladdin. Tetrabutylammonium bromide (99%) and super dried acetonitrile (THF and DMSO, water ≤30 ppm) were obtained from J&K Chemicals Co. (Beijing, China). All chemicals and solvents were directly used without further purification unless noted.

^1^H NMR spectra was recorded on a Bruker AV400 with tetramethylsilane as internal standard. Fourier transform infrared spectroscopy (FTIR) was collected on Frontier Mid-IR FTIR (Perkin Elmer). X-ray photoelectron spectra (XPS) were recorded on ESCALAB250Xi (Thermo Scientific, Waltham, MA, USA). TEM was performed on HITACHI-HT7700 TEM instruments with an accelerating voltage of 100 kV. Dynamic light scattering (DLS) was recorded on Zetasizer Nano ZS90. Fluorescence spectra were carried out on a F4600 FL (Hitachi, Chiyoda ku, Japan) spectrofluorometer with xenon discharge lamp excitation. Cells were imaged on a confocal microscope (Olympus FV1000-IX81). All images were analyzed with Olympus FV1000-ASW.

### 2.2. Synthesis of HNPs with Different Shapes

Spindle HNPs were prepared according to our previous work [38]. Typically, Fe(NO_3_)_3_·9H_2_O (4.04 g, 0.01 mol) was dissolved in 250 mL distilled water. Under stirring, 1 M NaOH were added to above solution until pH was about 10.5–10.8. Then the supernatant was removed and washed by distilled water several times until pH was about 9.3. After that, 200 mL distilled water was added to the solution. Under stirring, 1 M HCl (20 mL) and 0.1 M NaH_2_PO_4_ (2.25 mL) were added to above 200 mL solution. Then the mixture was transferred into 500 mL flask and added water to 500 mL. The system was placed in an oil bath for 2–3 days at 100 ± 2 °C until solution changed into brick-red color. The mixture was centrifuged and precipitate was washed with distilled water for three times. Elliptical and quasi-spherical α-Fe_2_O_3_ nanoparticles (named as ENP and QSNP, respectively) were prepared by the similar procedure except adding different amounts of 0.1 M NaH_2_PO_4_ (1 mL for elliptical ones, and 0.1 mL for quasi-spherical ones, respectively).

### 2.3. Adsorption of PVP

Polyvinyl pyrrolidone (PVP) (7.2 g) were dissolved into 130 mL distilled water under sonication. The HNPs (0.6 g) were then dispersed in above solution and sonicated for 20–30 min. The mixture was stirred for 24 h at room temperature with mechanical stirring for the rotate speed with 400 rpm. The nanoparticles were centrifuged and precipitate was washed with distilled water for three times.

### 2.4. Fabrication of Fluorescent HNPs

Fluorescent HNPs were prepared via a typical sol-gel method. Generally, 10 mg PVP-coated HNPs as above were dispersed in a solution of ethanol (32 mL), ammonium hydroxide (1 mL) and distilled water (8 mL) and the mixture was sonicated for 20 min. AIEgen-Si(OCH_3_)_3_ (10 mg) was dissolves in DMSO (0.2 mL) in a small centrifuge tube, then 1 mL ethanol and 0.05 mL of TEOS was added into the above solution. The mixed solution was added to PVP-coated HNPs suspension drop by drop. Then the mixture was stirred for 24 h at room temperature. The nanoparticles were centrifuged and precipitate was washed with distilled water and ethanol each for three times.

### 2.5. Cell Viability

The cell cytotoxicity of fluorescent HNPs were assessed in HeLa cells using a standard 3-(4,5-dimethythiazol-2-yl)-2,5-diphenyltetrazolium bromide (MTT) assays. In details, the cells were seeded in 96-well plates at a density of 7 × 10^3^ cells/well in a 5% CO_2_ incubator at 37 °C. After overnight, the cells were incubated with 20, 40, 60, 100, 160 μg/mL fluorescent HNPs for 24 h; 10 μL of MTT (5 mg/mL) dye was added to each well and incubated for 4 h at room temperature. Then the upper solution was carefully removed and 150 μL of DMSO was added to each well. The plate was gently shaken for 30 min and analyzed with a plates reader (Spectra MAX 340PC). The absorbance of purple formazan at 492 nm was monitored.

### 2.6. Cell Imaging

HeLa cells were seeded onto six-well plates at a density of 2.5 × 10^5^ cells/well in a 5% CO_2_ incubator at 37 °C. Then 2 mL fluorescent HNPs at a concentration of 10 µg/mL in cell culture medium (DMEM + fetal bovine serum (FBS) + Penicillin-Streptomycin (*v*/*v*/*v* = 100:10:1)) was added to the well and incubated for various times at 37 °C. Then the cells were washed thrice with PBS (pH 7.0–7.2) to remove free fluorescent HNPs and directly observed by CLSM. Cells images were obtained by confocal laser scanning microscope (Olympus FV1000-IX81) with the excitation and emission wavelength of 405 and 490–550 nm, respectively.

## 3. Results and Discussion

### 3.1. Preparation of HNPs with Different Shapes

**Figure 1:**
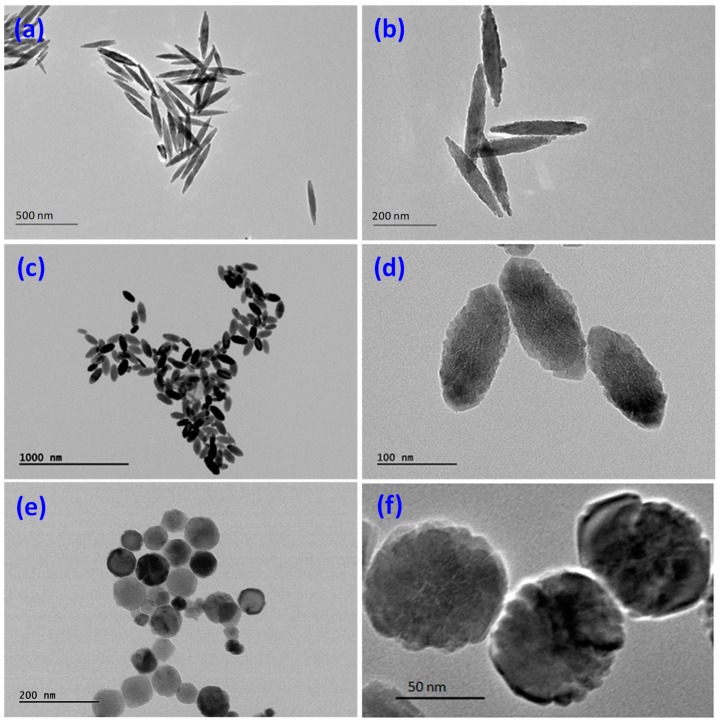
TEM images of α-Fe_2_O_3_ nanoparticles (HNPs) with three different shapes. (**a**,**b**) spindle shape; (**c**,**d**) ellipsoidal shape; (**e**,**f**) quasi-spherical shape. (**b**,**d**,**f**) are TEM images at high magnification of (**a**,**c**,**e**), respectively.

The α-Fe_2_O_3_ nanoparticles (HNPs) with three different shapes, i.e., spindle shape, ellipsoidal shape, and quasi-spherical shape, can be easily obtained via a conventional hydrothermal method under appropriate temperature and pH as reported in the literature [39,40]. Figure 1 shows TEM images of the as-prepared HNPs. From Figure 1, it was observed that the spindle shape HNPs had narrow size distribution with measured dimensions of 354 ± 70 nm for the longer axis and 56 ± 8 nm for the shorter axis with an aspect ratio of 5.92–6.63 (Figure 1a,b). The long axis of the ellipsoidal HNPs was 160 ± 10 nm and the short axis was 70 ± 8 nm with an aspect ratio of 2.20-2.42 (Figure 1c,d), respectively. The average diameter of quasi-spherical HNPs is about 84 ± 10 nm.

### 3.2. Reaction Condition Optimization

**Scheme 1:**
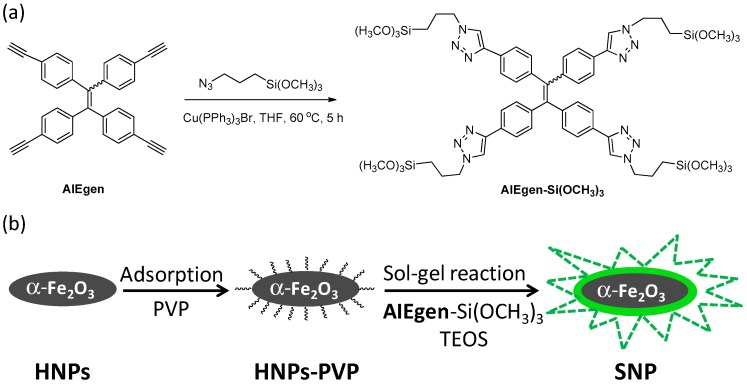
Representative preparation route of fluorescent HNPs taking the spindle one (SNP) as an example.

The synthetic route of fluorescent HNPs was illustrated in Scheme 1 taking the spindle one (SNP) as an example. The functionalized AIEgen derivatives (AIEgen-Si(OCH_3_)_3_) was first synthesized by copper-catalyzed click reaction of 3-azidopropyltrimethoxysilane with 1,1,2,2-tetrakis(4-ethynylphenyl)-ethene to produce AIEgen-Si(OCH_3_)_3_ with a five-membered heteroatom spacer [31]. FTIR spectra was used to monitor the formation of AIEgen-Si(OCH_3_)_3_. As shown in Figure S1, both the strong band at 2100 cm^−1^ assigned to –N≡N– group of 3-azidopropyltrimethoxysilane and the characteristic bands at 3294 cm^−1^ and 2114 cm^−1^ corresponding to stretching vibrations of C≡C–H and C≡C groups in the AIEgen, respectively, disappeared in the spectra of AIEgen-Si(OCH_3_)_3_ (c line), indicating complete reaction of four C≡C in the AIEgen to produce the target compound, AIEgen-Si(OCH_3_)_3_.

**Figure 2:**
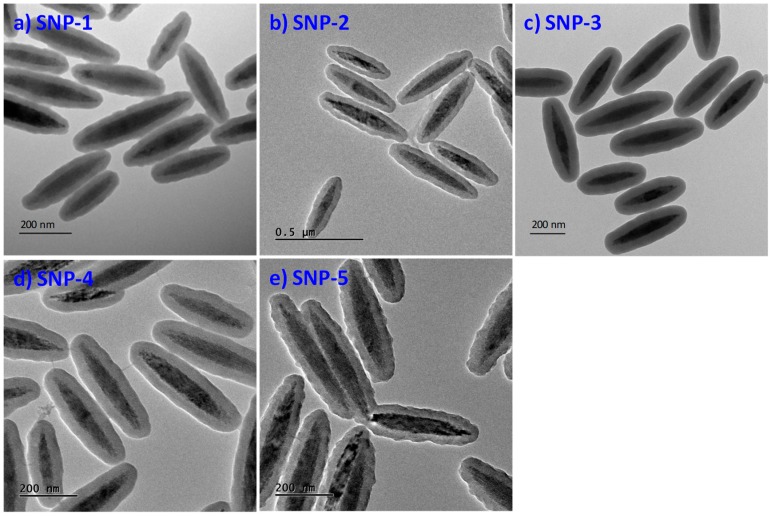
TEM images of fluorescent α-Fe_2_O_3_ NPs with spindle shape prepared according the reaction conditions listed in Table 1 (Entries 1–5).

Before the sol-gel reaction, polyvinyl pyrrolidone (PVP) was adsorbed onto HNPs’ surfaces to enhance their stability and dispersion in aqueous solution [41]. Subsequently, AIEgen-Si(OCH_3_)_3_ and TEOS with given ratios were added into the mixture of ethanol, ammonium hydroxide and distilled water in the presence of PVP-coated HNPs. After the so-gel reaction, fluorescent HNPs was achieved by capturing AIEgens inside thin SiO_2_ shells around HNPs. Due to the high brightness and good photostability of fluorescent NPs being of importance for bio-imaging application, reaction conditions for preparation of fluorescent HNPs were carefully optimized by adjusting the concentrations of AIEgen-Si(OCH_3_)_3_ and TEOS in feed. Table 1 summarizes the reaction conditions and the properties of the as-prepared fluorescent HNPs. The corresponding TEM images are also shown in Figure 2. The chemical compositions of the resultant products were determined by FTIR and XPS spectra as shown in Figures S2 and S3.

Firstly, the effect of concentration of TEOS in feed on the morphology and optical properties of α-Fe_2_O_3_ NPs was carefully investigated. As shown in Figure 2a–c and Table 1 (entries 1–3), as the amounts of TEOS increased from 0.05 mL to 0.20 mL in feed, the thickness of the silica shell on the surface of α-Fe_2_O_3_ NPs gradually increased from 26 to 54 nm. The results agreed with those reported in the literature [42,43]. The aspect ratio of the resultant fluorescent HNPs became smaller, while their average size became larger compared to the original HNPs (Table 1).

In addition, it was observed that the amounts of AIEgen-Si(OCH_3_)_3_ in feed only slightly affected the sol-gel reactions as shown in Figure 2b,d,e and Table 1 (entries 2, 4–5). From Figure 2b,d,e, it was obvious that every HNP was uniformly coated by a silica shell layer, but there was no significant change in thickness of the silica shell on the surface of the resultant fluorescent HNPs when the amounts of AIEgen-Si(OCH_3_)_3_ increased from 4.5 to 20 mg. Moreover, from TEM images, it was interesting to note that multicore NPs and secondary nucleation of small silica colloids did not occur in the process of sol-gel reactions, thus, only single HNPs were decorated with AIEgens, which was different from the case reported by Tang and coworkers [32], where several magnetite Fe_3_O_4_ nanoparticles with smaller size aggregated with each other to form the multiple Fe_3_O_4_ cores when the sol-gel reaction was going on. This result can be explained as follows: Prior to the sol-gel reaction, HNPs were stabilized by PVP very well, resulting in the silica growing directly onto the adsorbed polymer [41] to prevent the formation of multinuclear NPs when the hydrolysis reaction occurred.

**Figure 3:**
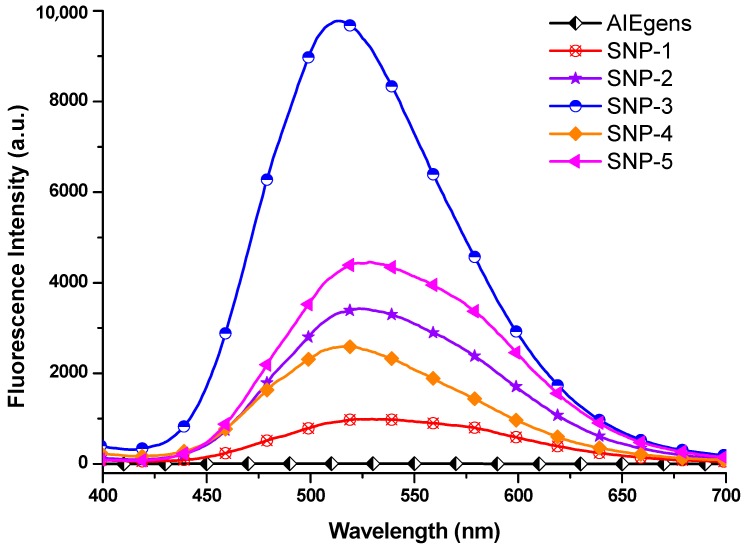
Fluorescence spectra of AIEgens in THF (concentration: 1 × 10^−3^ mol/L) and fluorescent HNPs with spindle shape (SNP) dispersed in water (concentration: 200 μg/mL). Excitation wavelength: 360 nm. SNP samples were prepared according to the reaction conditions listed in Table 1.

Figure 3 shows the fluorescence spectra of AIEgens in THF (1 × 10^−3^ mol/L) and the as-prepared fluorescent α-Fe_2_O_3_ NPs with spindle shape (SNP) dispersed in water with the concentration of 200 μg/mL. From Figure 3, it was observed that AIEgens themselves were almost non-emissive in solution due to non-radiative energy loss caused by the rotational and vibrational motions of their phenyl rings. Once they were captured into the SiO_2_ shell on the surface HNPs, they exhibited strong fluorescent emission centered at about 510 nm upon excitation at 360 nm. Furthermore, it was noted that the thicker the silica shell, the higher the fluorescence quantum yield (*Φ*) of AIEgens inside them (entries 1–3, Table 1), which should be attributed to the restrictions of intramolecular rotations of AIEgens in the aggregate state [44]. In addition, the fluorescence quantum yield (*Φ*) significantly increases with the increase of AIEgens amounts in feed when TEOS content is constant (entries 2, 4, 5, Table 1). Clearly, the fluorescence quantum yields of all tested samples are relatively low compared with those of silica hybrid nanoparticles, in which 1,1,2,2-tetraphenylethene derivatives were covalently immobilized (*Φ* is about 10~40%) [30,31]. These results should be attributed to the thin silica shell on the surface of α-Fe_2_O_3_ nanoparticles which could not effectively inhibit the rotational and/or vibrational motions of AIEgens within it, resulting in non-radiation energy consumption. However, rather than common organic dyes, the fluorescence quantum yields of our samples are very competitive and could meet the requirements of biological imaging. Since the purpose of preparing fluorescent inorganic NPs in this work is to address the relationship between their morphology and the interaction with living cells, both maintained original morphology of nanoparticles as far as possible and the suitable fluorescence brightness was desired. Along this line, the reaction conditions with 0.1 mL of TEOS and 10 mg AIEgens were chosen for the subsequent preparation of other fluorescent HNPs with different shapes.

### 3.3. Preparation of Fluorescent HNPs with Different Shapes

**Figure 4:**
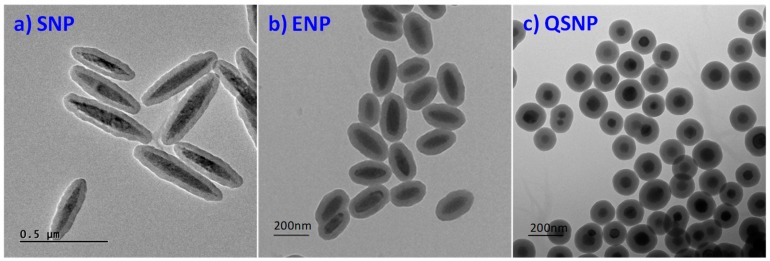
TEM images of fluorescent HNPs with different shapes.

According to the optimized reaction conditions, three fluorescent HNPs with different shapes, i.e., spindle shape (SNP), ellipsoidal shape (ENP), and quasi-spherical shape (QSNP), were obtained, respectively. Their TEM images are shown in Figure 4 and the properties are summarized in Table 2.

From Figure 4, it was obvious that the as-prepared fluorescent HNPs maintained the original shapes of HNP templates except for the decreased aspect ratios for SNP and ENP samples. Besides, some fluorescent HNPs containing several HNP cores existed in the QSNP sample (Figure 4c). This phenomenon could be explained as follows: compared with the colloid diameter of the quasi-spherical HNPs (84 ± 10 nm), the PVP with the average *M*_w_ of 58,000, which was coated on the surface of HNPs prior to the sol-gel reaction appeared to be too large to sufficiently stabilize the HNPs. Thus, sometimes, some HNPs would aggregate together in the process of StÖber growth, similar to the case of silica-covered gold NPs reported in the literature [41].

**Figure 5:**
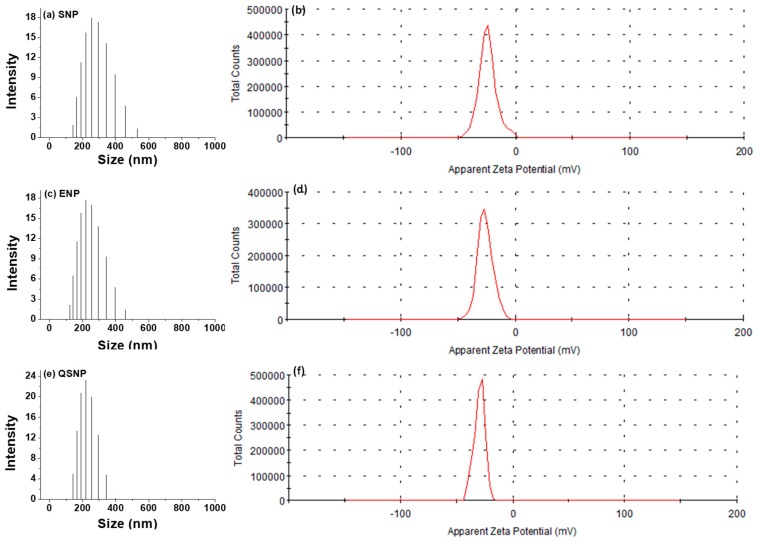
Hydrodynamic diameter distribution (**a**,**c**,**e**) and Zeta potentials (**b**,**d**,**f**) of the as-prepared fluorescent HNPs: (**a**,**b**) for SNP; (**c**,**d**) for ENP; (**e**,**f**) for QSNP. All samples are dispersed in water with concentration of 200 μg/mL.

Dynamic light scattering measurements were performed to determine the hydrodynamic diameters and Zeta potentials of the resultant fluorescent HNPs. As shown in Figure 5 and Table 2, average hydrodynamic diameters for three samples were 258 nm for SNP, 221 nm for ENP, and 217 nm for QSNP, with the corresponding narrow polydispersity index (PDI) of 0.075, 0.074, and 0.01, respectively. Further, colloidal stability is an important and fundamental parameter for NPs for their various applications, which can be evaluated by surface charges or zeta potential [45]. From Figure 5b,d,f and Table 2, the zeta potentials of three fluorescent HNPs with different shapes were −24.3 mV for SNP, −26.1 mV for ENP, and −29.6 mV for QSNP, respectively. The zeta potentials of three samples in other mediums containing fetal bovine serum (FBS) were determined according to the similar method (Table S1 and Figure S4). These results implied good stabilities of three samples in a physiological environment.

**Figure 6:**
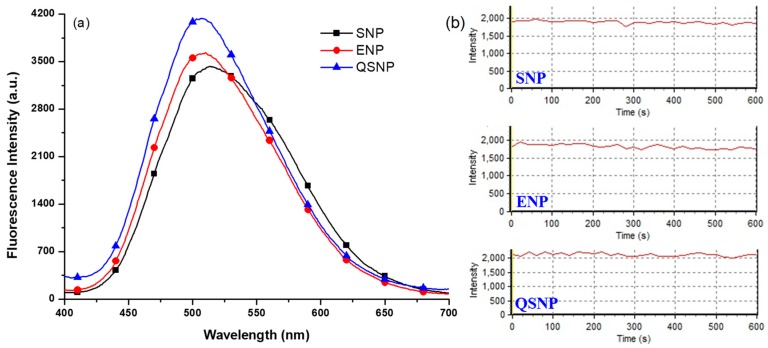
(**a**) Fluorescence spectra of three NPs with different shapes dispersed in water (concentration: 200 μg/mL). Excitation wavelength: 360 nm. (**b**) Time courses of fluorescent intensities at 510 nm of three samples. The sample concentration is 10 μg/mL in cell culture medium (DMEM/fetal bovine serum (FBS)/Penicillin-Streptomycin = 100:10:1, *v*/*v*/*v*). 2 mL of samples were used in the study. *λ*_ex_ = 405 nm.

Figure 6a shows the photoluminescence spectra of three fluorescent HNPs dispersed in water with the same concentration of 200 μg/mL. They exhibited similar spectral shapes and intense fluorescence emission. To assess the photostability of three samples, photobleaching experiments were carried out by monitoring their fluorescent intensity at 510 nm in cell culture medium over a period of 600 s (Figure 6b) using laser as an excitation source. As shown in Figure 6b, the output of the fluorescence signal was very stable under a continuous 405nm laser beam irradiation for 600 s under confocal microscope and there was no significant photobleaching for three samples, indicating that these fluorescent HNPs could be used as probes to long-term-label cells with good photostability.

### 3.4. Cell Viability

**Figure 7:**
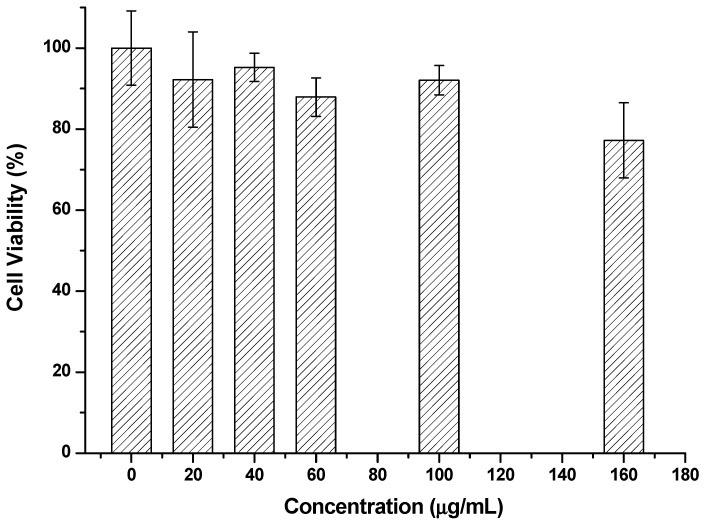
Cell viability of Hela cells treated with different concentrations of SNP for 24 h.

The biocompatibility of fluorescent HNPs is crucial for their biomedical applications [46]. The cytotoxicity of fluorescent HNPs against Hela cells was examined by a widely used MTT assay. Here, only the result of spindle fluorescent HNPs (SNP) was presented in Figure 7 as an example, since three fluorescent samples had the same chemical compositions. From Figure 7, compared with the control, the cell viabilities of Hela cells suffered the treatment with the SNP at various concentrations exhibiting slight changes in the range of 0–100 μg/mL for 24 h. Even when the concentration of SNP was increased to 160 μg/mL, the cell viability was still higher than 77%. These results demonstrated the low toxicity of SNP to living cells.

### 3.5. Cell Imaging

**Figure 8:**
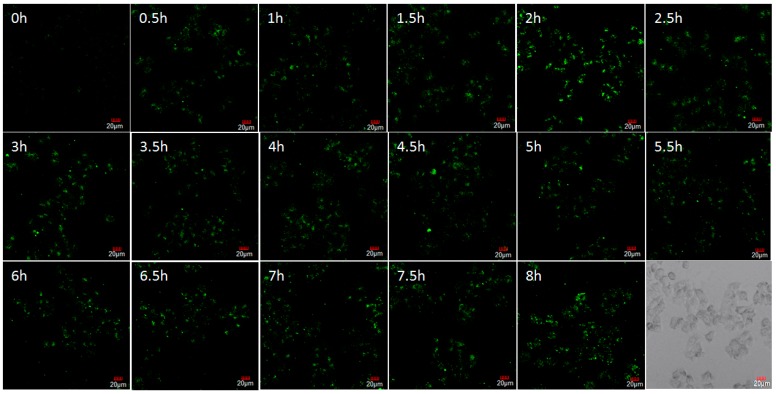
CLSM images of HeLa cells after incubation with SNP (10 μg/mL) for different times. The last one is the bright field image of HeLa cells, corresponding to CLSM images with incubation time of 2 h.

**Figure 9:**
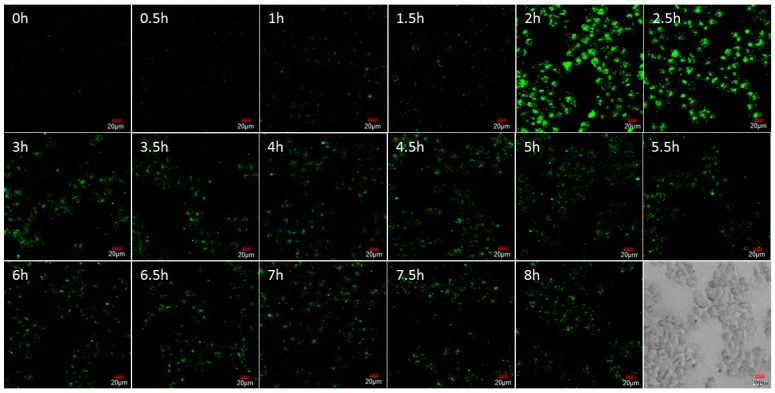
CLSM images of HeLa cells after incubation with ENP (10 μg/mL) for different times. The last one is the bright field image of HeLa cells, corresponding to CLSM images with incubation time of 2 h.

**Figure 10:**
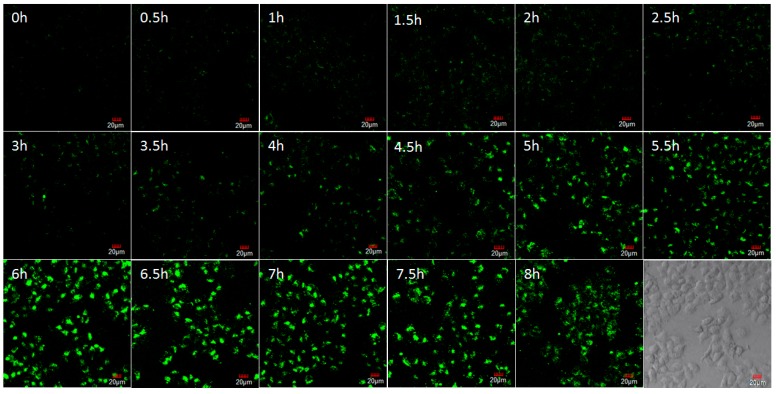
CLSM images of HeLa cells after incubation with QSNP (10 μg/mL) for different times. The last one is the bright field image of HeLa cells, corresponding to CLSM images with incubation time of 6 h.

**Figure 11:**
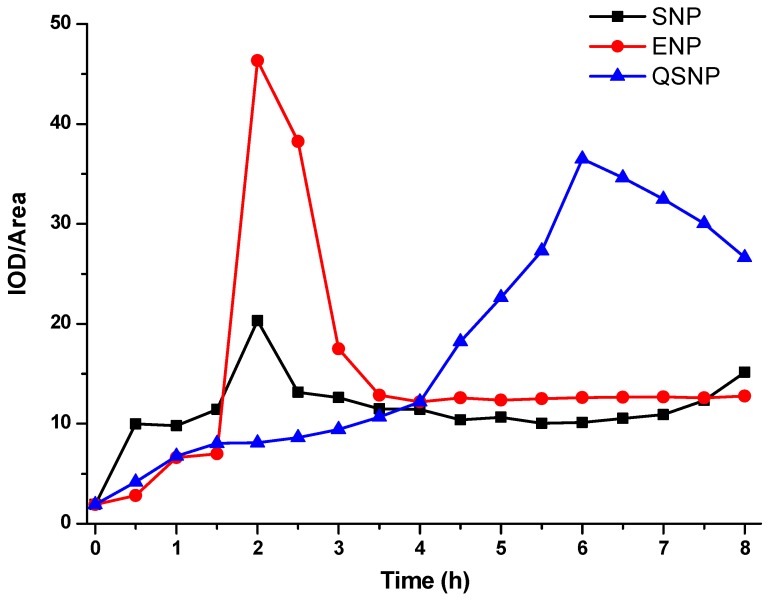
Variations of the average optical density (IOD/Area) with times. The analyzed number of cells is 20.

Three fluorescent HNPs with different shapes, i.e., SNP, ENP, and QSNP were co-cultured with Hela cells for various times to preliminarily explore the influence of their morphologies on the interaction with cells. In this experiment, 2 mL of sample with the concentration of 10 μg/mL in cell culture medium was added to the Hela cells and incubated for various times at 37 °C. After washing the cells with PBS buffer, the cells were directly observed by CLSM. Figure 8, Figure 9 and Figure 10 show the representative CLSM images of SNP, ENP, and QSNP at different times, respectively. From Figure 8, Figure 9 and Figure 10, three samples could be gradually taken in the cytoplasm with the extension of incubation time, but, time-dependent cell uptake behaviors for three samples were significantly different (Figure 11). The average optical density (IOD/area) values were extracted from CLSM images based on 20 cells by Image-J. From Figure 11, the IOD/area values for three samples were gradually increasing at the beginning, reaching the maximum, then dropping until to a certain value which was possibly due to the fluorescent nanoparticles being excluded from cellular internalization altogether when the size of any particle exceeded 150 nm [47,48]. The larger amount ENP seemed be taken into cells at a similar rate compared to that of SNP since they had approximate fluorescence quantum yield (*Φ*) (Table 2). The IOD/area of ENP sample reached a maximum after incubation with Hela cells for 2 h. This result was consistent with the case of ellipsoidal mesoporous silica NPs with an aspect ratio of 1.51–2.39 reported in the literature [10]. In the case of QSNP, the IOD/area peak was reached when the incubation time was extended to 6 h, implying its lowest cellular uptake rate under the same conditions compared to those of the other two tested samples with different shapes.

## 4. Conclusions

In conclusion, we described a facile and general method to fabricate fluorescent inorganic nanoparticles by decorating AIEgens around non-luminescent nanoparticles. In this method, the AIEgens were captured into the thin SiO_2_ layer on the surface of inorganic nanoparticles by the sol-gel reaction. According to the strategy, three fluorescent α-Fe_2_O_3_ nanoparticles with different shapes were achieved as a proof-of-concept system. They exhibited intense green fluorescence in water upon excitation at 360 nm with high fluorescence quantum yield. In addition, the as-prepared fluorescent nanoparticles showed low cytotoxicity against living cells even at concentrations up to 160 μg/mL. They could be effectively internalized by HeLa cells. The time-dependent CLSM imaging experiments demonstrated that the shapes of NPs had a great effect on cellular endocytosis, and ellipsoidal NPs exhibited the highest uptake rate under the same experimental conditions. This work should open a novel way to fabricate various fluorescent inorganic nanoparticles with specific morphology for diverse biological applications.

## Figures and Tables

**Table 1:** Screening of reaction conditions and the properties of the fluorescent α-Fe_2_O_3_ NPs ^a^.

Entry	Sample Name	TEOS (mL)	AIEgens (mg)	Thickness of SiO_2_ Shell (nm) ^b^	Aspect Ratio ^b^	*λ*_em_^c^	*Φ*_F_^d^ (%)
1	SNP-1	0.05	10	26	3.51–3.76	520	3.12
2	SNP-2	0.10	10	32	3.41–3.71	514	4.74
3	SNP-3	0.20	10	54	2.68–2.89	504	6.42
4	SNP-4	0.10	4.5	30	3.18–3.53	506	2.56
5	SNP-5	0.10	20	40	3.35–3.75	518	9.94

^a^ AIEgen-Si(OCH_3_)_3_ and TEOS was added into the mixed solution of ethanol (32 mL), ammonium hydroxide (1 mL) and distilled water (8 mL) in the presence of 10 mg of PVP-coated HNPs. ^b^ Determined by TEM. ^c^ Excitation wavelength = 360 nm. ^d^ Determined by using rhodamine B (*Φ* = 0.73, in ethanol) as the fluorescence standard.

**Table 2:** The properties of the fluorescent α-Fe_2_O_3_ NPs with different shapes ^a^.

Sample	Thickness of SiO_2_ Shell (nm) ^b^	Aspect Ratio ^b^	Hydrodynamic Diameter (nm) ^c^	Zeta Potential (mV) ^c^	*λ*_em_^d^	*Φ*_F_^e^ (%)
SNP	32	3.41–3.71	258	−24.3	514	4.74
ENP	39	1.65–1.81	221	−26.1	507	5.77
QSNP	43	1.05–1.07	217	−29.6	507	2.87

^a^ AIEgen-Si(OCH_3_)_3_ (10 mg) and TEOS (0.1 mL) was added into the mixed solution of ethanol (32 mL), ammonium hydroxide (1 mL) and distilled water (8 mL) in the presence of 10 mg of PVP-coated HNPs. ^b^ Determined by TEM. ^c^ Measured by DLS. ^d^ Excitation wavelength = 360 nm. ^e^ Determined by using rhodamine B (*Φ* = 0.73, in ethanol) as the fluorescence standard.

## Supplementary Materials

The following are available online at http://www.mdpi.com/2079-4991/9/2/154/s1. Synthesis of AIEgen-Si(OCH_3_)_3_ and determination of fluorescence quantum yield; Figure S1: FTIR spectra of AIEgen-Si(OCH_3_)_3_; Figure S2: FT-IR spectra of SNP-1 sample; Figure S3: XPS spectra SNP-1 sample.

## References

[1] Champion J.A., Katare Y.K., Mitragotri S. (2007). Particle shape: A new design parameter for micro-and nanoscale drug delivery carriers. J. Control. Release.

[2] Mitragotri S., Lahann J. (2009). Physical approaches to biomaterial design. Nat. Mater..

[3] Canelas D.A., Herlihy K.P., DeSimone J.M. (2009). Top-down particle fabrication: Control of size and shape for diagnostic imaging and drug delivery. Wiley Interdiscip. Rev. Nanomed. Nanobiotechnol..

[4] Champion J.A., Mitragotri S. (2009). Shape induced inhibition of phagocytosis of polymer particles. Pharm. Res..

[5] Bartczak D., Muskens O.L., Nitti S., Sanchez-Elsner T., Millar T.M., Kanaras A.G. (2012). Interactions of human endothelial cells with gold nanoparticles of different morphologies. Small.

[6] Wang C., Ito Y., Pradeep B., Valiyaveettil S. (2015). Shape sensitivity on toxicity of gold nanoplates in breast cancer cells. J. Nanosci. Nanotechnol..

[7] Huang X., Teng X., Chen D., Tang F., He J. (2010). The effect of the shape of mesoporous silica nanoparticles on cellular uptake and cell function. Biomaterials.

[8] Meng H., Yang S., Li Z., Xia T., Chen J., Ji Z., Zhang H., Wang X., Lin S., Huang C. (2011). Aspect ratio determines the quantity of mesoporous silica nanoparticle uptake by a small gt pase-dependent macropinocytosis mechanism. ACS Nano.

[9] Huang X., Li L., Liu T., Hao N., Liu H., Chen D., Tang F. (2011). The shape effect of mesoporous silica nanoparticles on biodistribution, clearance, and biocompatibility in vivo. ACS Nano.

[10] Hao N., Li L., Zhang Q., Huang X., Meng X., Zhang Y., Chen D., Tang F., Li L. (2012). The shape effect of PEGylated mesoporous silica nanoparticles on cellular uptake pathway in Hela cells. Microporous Mesoporous Mater..

[11] Safi M., Yan M., Guedeau-Boudeville M.-A., Conjeaud H., Garnier-Thibaud V., Boggetto N., Baeza-Squiban A., Niedergang F., Averbeck D., Berret J.-F. (2011). Interactions between magnetic nanowires and living cells: Uptake, toxicity, and degradation. ACS Nano.

[12] Cardillo D., Tehei M., Hossain M.S., Islam M.M., Bogusz K., Shi D., Mitchell D., Lerch M., Rosenfeld A., Corde S. (2016). Synthesis-dependent surface defects and morphology of hematite nanoparticles and their effect on cytotoxicity in vitro. ACS Appl. Mater. Interace.

[13] Boonrungsiman S., Suchaoin W., Chetprayoon P., Viriya-Empikul N., Aueviriyavit S., Maniratanachote R. (2017). Shape and surface properties of titanate nanomaterials influence differential cellular uptake behavior and biological responses in THP-1 cells. Biochem. Biophys. Rep..

[14] Geng Y., Dalhaimer P., Cai S., Tsai R., Tewari M., Minko T., Discher D.E. (2007). Shape effects of filaments versus spherical particles in flow and drug delivery. Nat. Nanotechnol..

[15] Zhang J., Xu B., Tian W., Xie Z. (2018). Tailoring the morphology of AIEgen fluorescent nanoparticles for optimal cellular uptake and imaging efficacy. Chem. Sci..

[16] Shukla S., Eber F.J., Nagarajan A.S., DiFranco N.A., Schmidt N., Wen A.M., Eiben S., Twyman R.M., Wege C., Steinmetz N.F. (2015). The impact of aspect ratio on the biodistribution and tumor homing of rigid soft-matter nanorods. Adv. Healthc. Mater..

[17] Moeller J., Luehmann T., Hall H., Vogel V. (2012). The race to the pole: How high-aspect ratio shape and heterogeneous environments limit phagocytosis of filamentous escherichia coli bacteria by macrophages. Nano Lett..

[18] Hickson J. (2009). In vivo optical imaging: Preclinical applications and considerations. Urol. Oncol..

[19] Liu J., Yang X., He X., Wang K., Wang Q., Guo Q., Shi H., Huang J., Huo X. (2011). Fluorescent nanoparticles for chemical and biological sensing. Sci. China Chem..

[20] Li K., Ding D., Zhao Q., Sun J., Tang B.Z., Liu B. (2013). Biocompatible organic dots with aggregation-induced emission for in vitro and in vivo fluorescence imaging. Sci. China Chem..

[21] Jaworska A., Wojcik T., Malek K., Kwolek U., Kepczynski M., Ansary A.A., Chlopicki S., Baranska M. (2015). Rhodamine 6G conjugated to gold nanoparticles as labels for both SERS and fluorescence studies on live endothelial cells. Microchim. Acta.

[22] Manjubaashini N., Thangadurai T.D., Bharathi G., Nataraj D. (2018). Rhodamine capped gold nanoparticles for the detection of Cr^3+^ ion in living cells and water samples. J. Lumin..

[23] Verma V.K., Tapadia K., Maharana T., Sharma A. (2018). Convenient and ultra-sensitive fluorescence detection of bovine serum albumin by using Rhodamine-6G modified gold nanoparticles in biological samples. J. Biol. Chem. Lumin..

[24] Seo S., Lee H.Y., Park M., Lim J.M., Kang D., Yoon J., Jung J.H. (2010). Fluorescein-functionalized silica nanoparticles as a selective fluorogenic chemosensor for Cu^2+^ in living cells. Eur. J. Inorg. Chem..

[25] Xu W., Park J.Y., Kattel K., Ahmad M.W., Bony B.A., Heo W.C., Jin S., Park J.W., Chang Y., Kim T.J. (2012). Fluorescein-polyethyleneimine coated gadolinium oxide nanoparticles as T-1 magnetic resonance imaging (MRI)-cell labeling (CL) dual agents. RSC Adv..

[26] Lee H.Y., Son H., Lim J.M., Oh J., Kang D., Han W.S., Jung J.H. (2010). BODIPY-functionalized gold nanoparticles as a selective fluoro-chromogenic chemosensor for imaging Cu^2+^ in living cells. Analyst.

[27] Yu X., Jia X., Yang X., Liu W., Qin W. (2014). Synthesis and photochemical properties of BODIPY-functionalized silica nanoparticles for imaging Cu^2+^ in living cells. RSC Adv..

[28] Karthik S., Puvvada N., Kumar B.N.P., Rajput S., Pathak A., Mandal M., Singh N.D.P. (2013). Photoresponsive coumarin-tethered multifunctional magnetic nanoparticles for release of anticancer drug. ACS Appl. Mater. Inter..

[29] Kim S., Pudavar H.E., Bonoiu A., Prasad P.N. (2007). Aggregation-enhanced fluorescence in organically modified silica nanoparticles: A novel approach toward high-signal-output nanoprobes for two-photon fluorescence bioimaging. Adv. Mater..

[30] Faisal M., Hong Y., Liu J., Yu Y., Lam J.W.Y., Qin A., Lu P., Tang B.Z. (2010). Fabrication of fluorescent silica nanoparticles hybridized with aie luminogens and exploration of their applications as nanobiosensors in intracellular imaging. Chem. Eur. J..

[31] Mahtab F., Lam J.W.Y., Yu Y., Liu J., Yuan W., Lu P., Tang B.Z. (2011). Covalent immobilization of aggregation-induced emission luminogens in silica nanoparticles through click reaction. Small.

[32] Mahtab F., Yu Y., Lam J.W.Y., Liu J., Zhang B., Lu P., Zhang X., Tang B.Z. (2011). Fabrication of silica nanoparticles with both efficient fluorescence and strong magnetization, and exploration of their biological applications. Adv. Funct. Mater..

[33] Xia Y., Li M., Peng T., Zhang W., Xiong J., Hu Q., Song Z., Zheng Q. (2013). In vitro cytotoxicity of fluorescent silica nanoparticles hybridized with aggregation-induced emission luminogens for living cell imaging. Int. J. Mol. Sci..

[34] Mao L., Liu M., Xu D., Wan Q., Huang Q., Jiang R., Shi Y., Deng F., Zhang X., Wei Y. (2017). Synthesis, surface modification and biological imaging of aggregation-induced emission (AIE) dye doped silica nanoparticles. Appl. Surf. Sci..

[35] Wang D., Chen J., Ren L., Li Q., Li D., Yu J. (2017). AIE gen-functionalised mesoporous silica nanoparticles as a FRET donor for monitoring drug delivery. Inorg. Chem. Front..

[36] Wang C., Li Q., Wang B., Li D., Yu J. (2018). Fluorescent sensors based on AIE gen-functionalised mesoporous silica nanoparticles for the detection of explosives and antibiotics. Inorg. Chem. Front..

[37] Li Q.-L., Wang D., Cui Y., Fan Z., Ren L., Li D., Yu J. (2018). AIEgen-functionalized mesoporous silica gated by cyclodextrin-modified CuS for cell imaging and chemo-photothermal cancer therapy. ACS Appl. Mater. Inter..

[38] Wang J., Zhu W., Liu L., Chen Y., Wang C. (2015). Synthesis and cellular internalization of spindle hematite/polymer hybrid nanoparticles. ACS Appl. Mater. Inter..

[39] Scheiner P., Ferric E. (1978). Hydrous oxide sols: III. Preparation of uniform particles by hydrolysis of Fe (III)-chloride,-nitrate, and-perchlorate solutions. J. Colloid Interface Sci..

[40] Ocana M., Morales M.P., Serna C.J. (1999). Homogeneous precipitation of uniform alpha-Fe_2_O_3_ particles from iron salts solutions in the presence of urea. J. Colloid Interface Sci..

[41] Graf C., Vossen D.L.J., Imhof A., van Blaaderen A. (2003). A general method to coat colloidal particles with silica. Langmuir.

[42] Lu Y., Yin Y., Li Z.-Y., Xia Y. (2002). Synthesis and self-assembly of Au@SiO_2_ core−shell colloids. Nano Lett..

[43] Zhang X., Niu Y., Li Y., Li Y., Zhao J. (2014). Preparation and thermal stability of the spindle α-Fe_2_O_3_@SiO_2_ core–shell nanoparticles. J. Solid State Chem..

[44] Jayaram D.T., Ramos-Romero S., Shankar B.H., Garrido C., Rubio N., Sanchez-Cid L., Borros Gomez S., Blanco J., Ramaiah D. (2016). In vitro and in vivo demonstration of photodynamic activity and cytoplasm imaging through TPE nanoparticles. ACS Chem. Biol..

[45] Fernandez-Nieves A., Nieves F.J.D. (1999). The role of zeta potential in the colloidal stability of different TiO_2_/electrolyte solution interfaces. Colloid Surf. A Physicochem. Eng. Asp..

[46] Foglia S., Ledda M., Fioretti D., Iucci G., Papi M., Capellini G., Lolli M.G., Grimaldi S., Rinaldi M., Lisi A. (2017). In vitro biocompatibility study of sub-5 nm silica-coated magnetic iron oxide fluorescent nanoparticles for potential biomedical application. Sci. Rep..

[47] Gratton S.E.A., Ropp P.A., Pohlhaus P.D., Luft J.C., Madden V.J., Napier M.E., DeSimone J.M. (2008). The effect of particle design on cellular internalization pathways. Proc. Natl. Acad. Sci. USA.

[48] Strobel C., Oehring H., Herrmann R., Foerster M., Reller A., Hilger I. (2015). Fate of cerium dioxide nanoparticles in endothelial cells: Exocytosis. J. Nanopart. Res..

