# Supplementary material for: Facile Fabrication of Fluorescent Inorganic Nanoparticles with Diverse Shapes for Cell Imaging
Source: Nanomaterials (Basel). 2019 Jan 26;9(2):154. doi: 10.3390/nano9020154 (PMC6410410; doi:10.3390/nano9020154)
Supplement: Supplementary file 1 [file nanomaterials-09-00154-s001.pdf]

Supporting Information

1. Synthesis of AIEgen-Si(OCH3)3

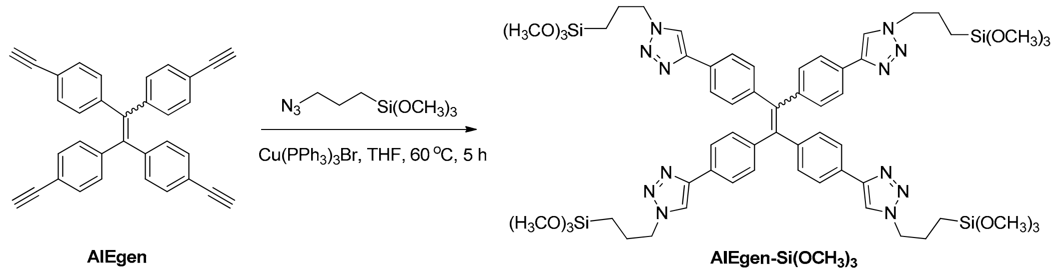

Scheme S1. Synthetic route of AIEgen-Si(OCH3)3.

1.1 Synthesis of the 3-azidopropyltrimethoxysilane

Synthesis of the 3-azidopropyltrimethoxysilane was according to the similar method in the literatures [1, 2]. In detail, 3-chloropropyltrimethoxysilane (2 mL, 10.875 mmol), sodium azide (1.76 g, 27.188 mmol), tetrabutylammonium bromide (0.644 g, 2 mmol) and dry acetonitrile (35 mL) were added to a 50 mL three-necked round-bottomed flask. Under N_2 atmosphere, the reaction mixture was stirred for 48 h at 82 °C. After the flask is cooled in air, the unreacted sodium azide was collected by centrifuged then used for recycling. The acetonitrile was removed under reduced pressure. About 15 mL cyclohexane was added to above mixture and subsequently filtered. Cyclohexane was removed from the filtrate under reduced pressure and the colorless liquid was obtained. ^{1}H NMR (400 MHz, $CDCl_3$), δ: 3.57 (s, 9H), 3.24-3.28 (t, 2H), 1.68-1.72 (m, 2H), 0.67-0.71 ppm (m, 2H).

1.2 Synthesis of AIEgen-Si(OCH3)3

AIEgen-Si(OCH3)3 was synthesized according to the literature [3]. In a 25 mL Schlenk tube, 17.34 mg (40.5 μmol) 1,1,2,2-tetrakis(4-ethynylphenyl)ethene and 33.25 mg (162 μmol) 3-azidopropyltrimethoxysilane and 9.04 mg (97.2 μmol) $Cu(PPh_3)_3Br$ were dissolved in 2 mL dry THF. After stirring for 5 h at 60 °C, the reaction mixture was centrifuged at 10000 rpm for 10 min. During the reaction, water and oxygen were carefully excluded. The THF was removed from supernatant under reduced pressure.

2. Additional spectra

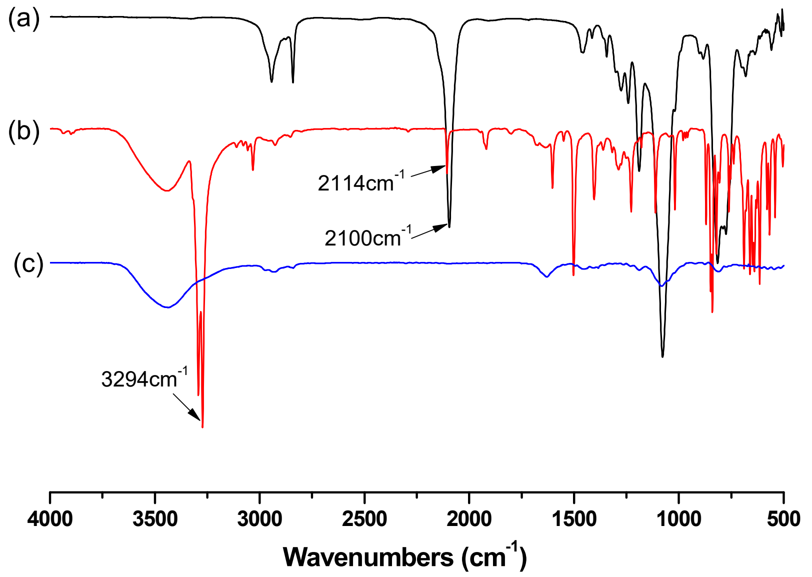

Figure S1. FTIR spectra of (a) 3-azidopropyltrimethoxysilane; (b) AIEgen; (c) AIEgen-$Si(OCH_3)_3$.

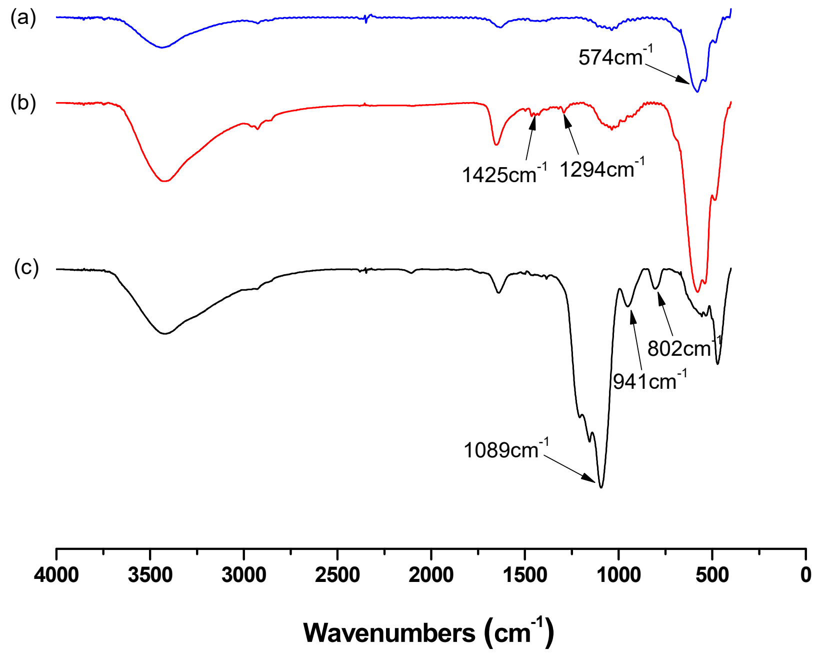

Figure S2. FTIR spectra of (a) HNPs; (b) PVP-coated HNPs; (c) SNP-1 sample prepared according to the reaction conditions listed in Table 1.

The chemical compositions of fluorescent α-Fe_2O_3 NPs were determined by

FTIR and XPS spectra taking the spindle fluorescent α-Fe_2O_3 NPs (SNP-1, Table 1) as an example. As shown in Figure S2, the typical bond related to the Fe-O stretches in α-Fe_2O_3 appears at 574 cm^{-1} (Figure S2a) [4]. The weak bands at 1294 and 1425 cm^{-1} ascribed to the stretching vibrations of N→H-O and pyrrole ring, respectively, were observed in the spectra of PVP-coated HNPs (Figure S2b) [5], demonstrating that PVP was adsorbed onto the surface of α-Fe_2O_3 NPs prior to sol-gel reaction. In the case of SNP-1 sample, intense absorption bands at 1089, 802 and 941 cm^{-1} assigned to the stretching vibrations of Si-O-Si and Si-OH appeared and the corresponding peak of Fe-O stretches in α-Fe_2O_3 at 574 cm^{-1} became weak as shown in Figure S2c [6], indicating that SiO_2 layer was formed around α-Fe_2O_3 NPs. It was noted that the characteristic peaks corresponding to AIE molecules could not be clearly observed, possibly due to their amounts captured in the SiO_2 layer were small.

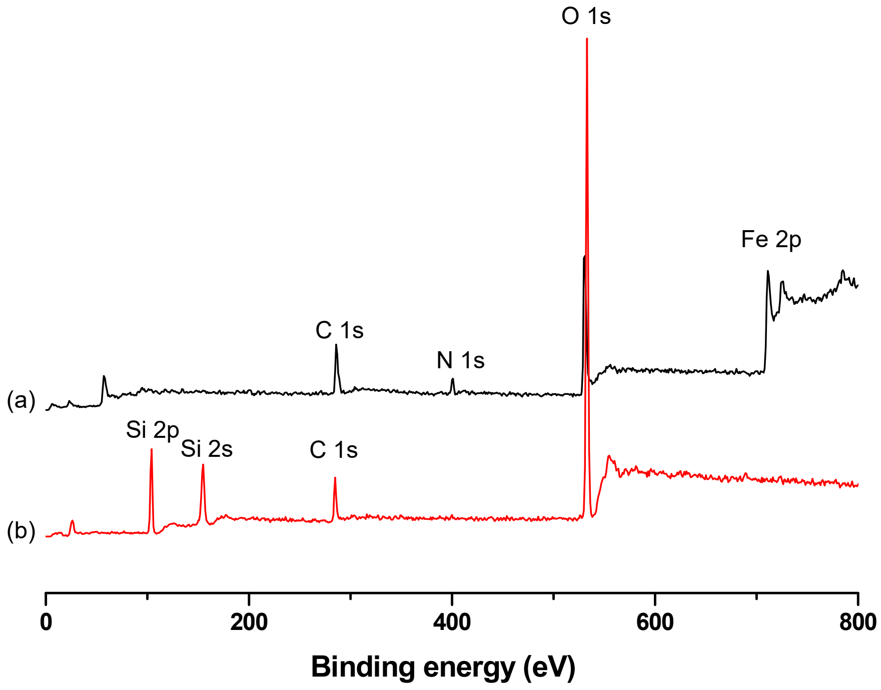

Figure S3. XPS spectra of PVP-coated HNPs (a); SNP-1 sample prepared according to the reaction conditions listed in Table 1 (b).

X-ray photoelectron spectra (XPS) of PVP-coated HNPs and fluorescent SNP-1

sample were depicted in Figure S3. There are four characteristic peaks at 285, 399, 529 and 710 eV in the XPS spectra of PVP-coated HNPs (Figure S3a) assigned to the binding energies of C1s, N1s, and O1s, and Fe2p, respectively, implying that PVP are covered around HNPs to prevent the aggregation of them each other via possibly main hydrogen bonding between N atoms in PVP and H-O groups on the surface of α-Fe_2O_3 NPs. For the fluorescent SNP-1 sample, its XPS spectra displays typical Si2s and Si2p peaks at 154 and 101 eV, respectively, but without Fe2p peaks. These results indicated that SiO_2 shell layer was successfully formed on the surface of α-Fe_2O_3 NPs, which covered up the characteristic peak of Fe2p due to the thickness of SiO_2 shell for SNP-1 sample was up to 26 nm.

3. Determination of fluorescence quantum yield

The fluorescence quantum yield of all as-prepared fluorescent HNPs samples were determined by using rhodamine B (Φ = 0.73, in ethanol) as the fluorescence standard, and were calculated according to the equation as following [7]:

$$\Phi_1 = \Phi_B \times \frac{\mathrm{Abs}_B \times F_1 \times \lambda_{exB} \times \eta_1}{\mathrm{Abs}_1 \times F_B \times \lambda_{ex1} \times \eta_B}$$

Subscripts 1 and B refer to the unknown sample and the standard sample, respectively. where Φ_1 is the fluorescence quantum yield, A is the absorbance at the excitation wavelength, F is the area under the corrected emission curve, and η is the refractive index of the solvent used.

4. Determination of Zeta potentials

Table S1 Zeta potentials of three fluorescent inorganic nanoparticles in different systems.[a]

Sample	Zeta potentials (mV)		
	Water	Water containing serum[b]	PBS buffer containing serum[c]
SNP	-24.3	-19.8	-9.73
ENP	-26.1	-20.0	-9.21
QSNP	-29.6	-18.1	-8.88

[a]Measured by DLS. [b]H_2O/fetal bovine serum (FBS) = 10:1 (v/v). [c]PBS buffer (0.0067 mol/L, pH 7.0-7.2) + fetal bovine serum (FBS) (PBS buffer/FBS = 10:1 (v/v)). [Sample] = 200 mg/mL.

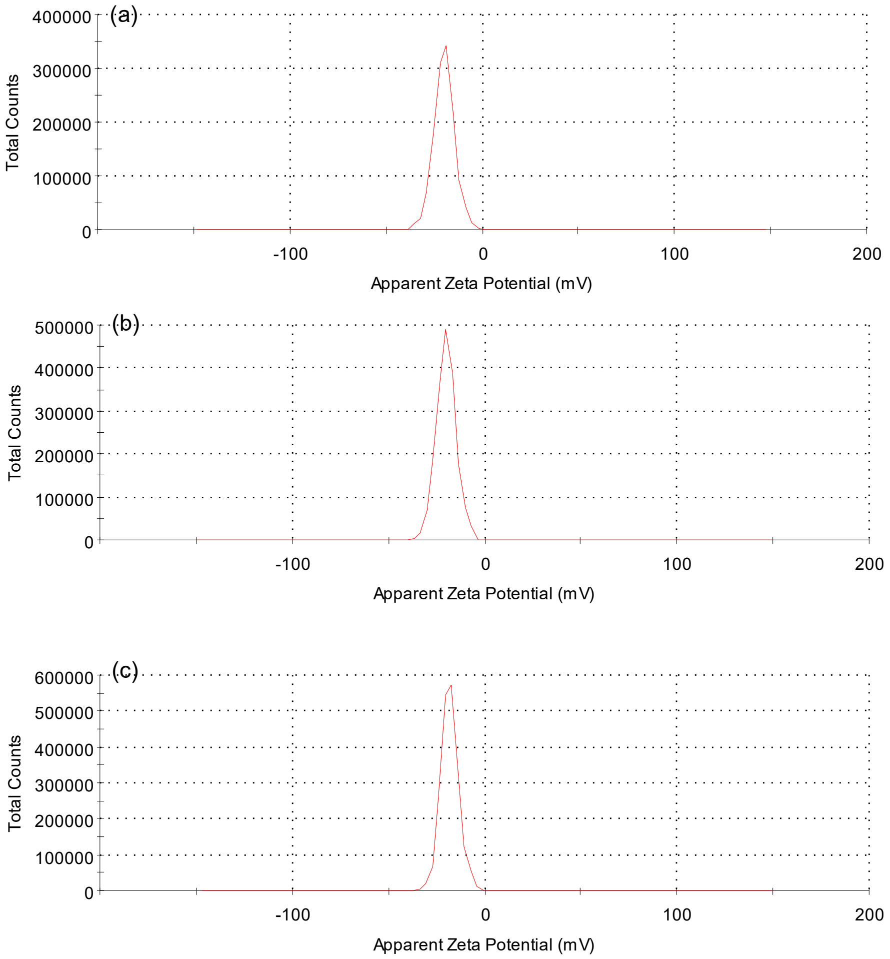

Figure S4. Zeta potentials of (a) SNP, (b) ENP and (c) QSNP in water containing fetal bovine serum (FBS) (H_2O/FBS = 10:1, v/v). The concentration of tested samples is 200 μg/mL.

References

1. Vilà, N.; André, E.; Ciganda, R.; Ruiz, J.; Astruc, D.; Walcarius, A. Molecular sieving with vertically aligned mesoporous silica films and electronic wiring through isolating nanochannels. *Chem. Mater.* **2016**, *28*, 2511-2514.
2. Lim, J.; Seong, L.S.; Ying, J.Y. Mesoporous silica-supported catalysts for metathesis: application to a circulating flow reactor. *Chem. Commun.* **2010**, *46*, 806-808.

3. Mahtab, F.; Lam, J.W.Y.; Yu, Y.; Liu, J.; Yuan, W.; Lu, P.; Tang, B.Z. Covalent immobilization of aggregation-induced emission luminogens in silica nanoparticles through click reaction. *Small* **2011**, *7*, 1448-1455.
4. Xuan, S.H.; Fang, Q.; Hao, L.; Jiang, W.; Gong, X.; Hu, Y.; Chen, Z. Fabrication of spindle Fe_2O_3@polypyrrole core/shell particles by surface-modified hematite templating and conversion to spindle polypyrrole capsules and carbon capsules. *Colloid Interf. Sci.* **2007**, *314*, 502-509.
5. Zhang, J.; Liu, H.; Wang, Z.; Ming, N. Au-induced polyvinylpyrrolidone aggregates with bound water for the highly shape-selective synthesis of silica nanostructures. *Chem.-Eur. J.* **2008**, *14*, 4374-4380.
6. Li, Q; Zhang, L.; Zhang, Z.; Zhou, N.; Cheng, Z.; Zhu, X. Air-tolerantly surface-initiated AGET ATRP mediated by iron catalyst from silica nanoparticles. *Polym. Sci. Polym. Chem.* **2010**, *48*, 2006-2015.
7. Karstens, T.; Kobs, K. Rhodamine B and rhodamine 101 as reference substances for fluorescence quantum yield measurements. *Phys. Chem.* **1980**, *84*, 1871-1872.
